# Nonselective Mevalonate Kinase Inhibitor as a Novel Class of Antibacterial Agents

**DOI:** 10.1155/2015/147601

**Published:** 2015-01-26

**Authors:** Mohammad Gharehbeglou, Ghasem Arjmand, Mohammad Reza Haeri, Mohammad Khazeni

**Affiliations:** ^1^Department of Medicine, Islamic Azad University, Qom Branch, Qom 37157, Iran; ^2^Department of Biochemistry, Payam Nour University of Mashhad, Mashhad 37157, Iran; ^3^Department of Biochemistry, School of Medicine, Qom University of Medical Science, Qom 37157, Iran; ^4^Booali Medical Research Center, Qom 37157, Iran

## Abstract

*Introduction.* There are a few evidences about targeting isoprenoids biosynthesis pathway in bacteria for finding new antibiotics. This study was conducted to assess antibacterial effects of vanadyl sulfate (VS), one of the mevalonate kinase inhibitors to find a new target for killing bacteria. *Materials and Methods*. Antibacterial effect of VS alone and in combination with glycine or EDTA was assessed on *Escherichia coli* and *Pseudomonas aeruginosa* as Gram-negative and *Staphylococcus aureus* and *Enterococcus faecalis* as Gram-positive bacteria using serial dilution method and minimum inhibitory concentrations (MICs) identified. *Result.* MICs for* S. aureus* and* E. coli* were 4 and 8 mg/mL, respectively. VS could not affect the growth of two other bacteria. However, VS in combination with glycine not only inhibited the growth of * E. faecalis* and * P. aeruginosa*, but also reduced MICs for VS-sensitive bacteria (*S. aureus* and* E. coli*). EDTA could reduce MIC for* E. coli * and * P. aeruginosa*. *Conclusion.* VS could inhibit the growth of * S. aurous* and * E. coli*, and adding glycine or EDTA improved VS antibacterial activity presumably via instability of the cell wall and enhanced transport of VS through bacterial cell wall. Inhibition of the isoprenoid pathway might provide new tools to overcome bacterial resistance.

## 1. Introduction

Isoprenoids are the largest class of small molecules in nature [1]. Ubiquinone, cholesterol [2, 3], carotenoid [4], and staphyloxanthin [5] are the representatives of isoprenoids which play several roles in living organisms. Isopentenyl diphosphate is synthesized via mevalonate and nonmevalonate pathway [6, 7]. In most bacteria, isoprenoids are produced by nonmevalonate pathway [8, 9]. However, a few bacteria (including some Gram-positive), fungi, and animals (including humans) synthesize their isoprenoid precursors using mevalonate pathway whereas others possess the two full pathways [9]. It is not surprising that some of the enzymes involved in isoprenoids biosynthesis could be targets for the development of novel antibacterial agents.

The inhibitory effect of small nontoxic amounts of vanadium salts (vanadyl sulfate) in the hepatic biosynthesis of cholesterol (a eukaryotic end product of isoprenoid pathway) has long been understood [10]. Recently it has been also reported that vanadyl sulfate can reduced serum cholesterol levels in patients with type 1 diabetes without any toxic effects [11]. It has been identified that vanadium reduces cholesterol biosynthesis by inhibiting the conversion of mevalonate to its phosphate and pyrophosphate esters [12]. Since the early steps of isoprenoid biosynthesis pathway (mevalonate kinase) in eukaryotes resemble bacterial isoprenoid biosynthesis and regarding the fact that VS inhibits the mevalonate pathway in eukaryotic organisms [10], we examined the effect of VS as a nonselective mevalonate kinase inhibitor on bacterial isoprenoid biosynthesis and its impact on bacterial growth. For this reason, two Gram-positive bacteria,* Staphylococcus aureus* and* Enterococcus Faecalis,* were selected. We also selected two Gram-negative bacteria which are not known to have mevalonate kinase (*Escherichia coli* and* Pseudomonas aeruginosa*) for making a comparison between two kinds of bacteria species and find if there is another site of action of VS other than mevalonate kinase. Our results show that although VS affect primarily mevalonate kinase dependent Gram-positive bacteria, it can reduce the growth of* E. coli* showing the presence of nonspecific action sites of VS.

## 2. Materials and Methods

Four kinds of bacteria were selected:* Staphylococcus aureus* (ATCC25923),* Enterococcus Faecalis* (ATCC 2599),* Escherichia coli* (ATCC 25922), and* Pseudomonas aeruginosa* (ATCC 27853). A suspension from each bacterium with turbidity equal to 0.5 McFarland was made with normal saline. MICs were assessed by serial dilution method using 96-well microplate [13]. Culture media (TSB), test compound, and a suitable amount of bacterial suspension were added to the wells to achieve a final concentration of bacteria at 5 × 10^5^. VS (Merck, Germany) was applied in two ways: alone and in combination with glycine or EDTA as a chelating agent. Firstly, different concentrations of VS dissolved in water (from 0.015 to 8 mg/mL) were used. In another set of experiments, VS was applied in equal molar concentration with glycine or EDTA. Two wells were considered as solvent and glycine controls in each experiment. Plates were incubated at 37°C for 24 hours. MIC was defined as the lowest concentration of antimicrobial agent that prevents visible growth of bacteria under an inverted microscope. For determination of the MLC, 10 *μ*L of each well that showed visible growth inhibition from the last positive one was streaked onto culture medium. After incubation at 37°C for 24 h, the MLCs were assessed visually as the lowest VS concentrations at which there was no bacterial growth. All the experiments were carried out in triplicate and the row data were statistically analyzed by *t*-test. Results with *P* ≤ 0.05 were considered statistically significant.

## 3. Results

VS significantly reduced the growth of* S. aureus* and* E. coli* in comparison with control. As shown in Table 1 the growth inhibition of* S. aureus* occurred at the concentration of 2 mg/mL (MIC = 2 and MLC = 4 mg/mL). Combination of VS with glycine did not affect MIC of VS on* S. aureus* but reduced MLC from 4 to 2 mg/mL. Antibacterial effect of VS on* E. coli* was observed at higher concentration (both MLC and MIC were 8 mg/mL). However, combination of VS and glycine could significantly reduce MIC from 8 mg/mL to 4 mg/mL (*P* = 0.018) and MLC from 8 mg/mL to 6 mg/mL (*P* > 0.05). Combination of VS with EDTA as a chelating agent could reduce MIC from 8 to 4 mg/mL (*P* < 0.05).

Interestingly, in spite of the fact that VS alone did not inhibit the growth of* E. faecalis*, application of VS with glycine could reduce the growth of bacteria (MLC = 8 and MIC = 2 mg/mL, *P* = 0.0001). VS did not show any inhibitory effect on* P. aeruginosa* at examined concentrations but VS with glycine or EDTA could reduce the growth of the bacteria. MICs were 2 and 4 mg/mL for glycine and EDTA, respectively (*P* < 0.05). Glycine per se did not show inhibitory effects on any of the bacteria.

## 4. Discussion

Curran and Costello reported the inhibitory effect of vanadyl salts on the biosynthesis of hepatic cholesterol [10]. Action site of vanadium on the biosynthesis of cholesterol demonstrated to be between mevalonate and its phosphate and pyrophosphate esters [12].

Considering the inhibitory effect of vanadyl salt on mevalonate kinase, there is a possibility that VS could inhibit growth of those bacteria with mevalonate kinase in their isoprenoid biosynthesis pathway. This study examined the antibacterial effect of VS through affecting mevalonate pathway of isoprenoid synthesis in bacteria.

As shown in Table 1, VS could reduce the growth of* S. aureus* with the lowest MIC (2 mg/mL). This indicates that VS could penetrate the peptidoglycan cell wall of the bacteria and exerts its inhibitory effect by targeting mevalonate kinase that could be the first target in* S. aureus*. However, VS could not affect the growth of Gram-positive* E. faecalis* at the highest concentrations used. The difference in VS action on two bacteria may be attributed to the difference in cell walls affecting transferring of VS across the cell wall. Interestingly, addition of glycine not only reduced the MIC of* E. faecalis* up to the level seen in* S. aureus*, but also reduced MLC for* S. aureus* from 4 mg/mL to 2 mg/mL (*P* = 0.0001) and so MIC became equal to MLC (Table 1). Since glycine inhibits cross-linking of peptidoglycan strands [14], it is concluded that glycine just facilitated the transport of VS across the cell wall. Therefore the absolute inhibitory activity is related to the VS.

It has been reported that many of Gram-negative bacteria like* E. coli* and* P. aeruginosa* do not have mevalonate pathway of isoprenoid synthesis [8, 9]. We selected two Gram-negative bacteria to find if there is another site of action of VS other than mevalonate kinase. If the mevalonate kinase is the only target of VS, the Gram-negative bacteria such as* E. coli* and* P. aeruginosa* must be resistant to VS. Vanadyl sulfate alone did not inhibit* P. aeruginosa* and inhibited* E. coli* only at high concentrations. Application of VS with glycine or EDTA in comparison with VS alone could considerably reduce MICs for* E. coli* and* P. aeruginosa*. The results indicate that Gram-positive bacteria are more sensitive to VS than Gram-negative bacteria, probably because of targeting the mevalonate kinase. However, inhibition of Gram-negative bacteria by VS even by adding glycine or EDTA shows that mevalonate kinase is not the only target of VS. It has been reported that vanadium interferes with phosphate metabolizing enzymes such as phosphatase [15], which may be an alternative target for mevalonate kinase in Gram-negative bacteria.

## 5. Conclusion

In summary, this study shows that VS is able to inhibit the growth of bacteria, especially Gram-positive bacteria, and this inhibitory effect could be amplified by adding glycine or EDTA as a chelating agent. Glycine is used in some washing up liquids, so VS could be added for the improvement of antiseptic activity. Furthermore, regarding the growing evidences about drug resistant pathogenic bacteria, hopefully this study results in more attention to the metabolic pathways that are less known as drug targets.

## Figures and Tables

**Table 1:** MIC and MLC of VS with or without glycine or EDTA.

	Vanadyl sulfate (mg/mL)		Vanadyl sulfate + glycine (mg/mL)		Vanadyl sulfate + EDTA (mg/mL)
	MIC	MLC	MIC	MLC	MIC
*S. aureus *	2	4	2	2	4
*E. faecalis *	—	—	2	8	—
*E. coli *	8	8	4	6	4
*P. aeruginosa *	—	—	2	2	4

Adding glycine or EDTA improved the antibacterial effect of  VS. Data are expressed as average of three assays.
